# Radiological Outcome of the Gradual Increase in Skeletal Traction in Acetabular Fracture

**DOI:** 10.7759/cureus.76725

**Published:** 2025-01-01

**Authors:** Haidar Nasuruddin, Mohd Shukri Omar, Aminudin Che Ahmad, Norhaslinda Bahaudin, Muhamad Syafiz Ahmad Ismani

**Affiliations:** 1 Orthopaedics, Traumatology and Rehabilitation, International Islamic University Malaysia, Kuantan, MYS; 2 Orthopaedics, Sultan Ismail Hospital, Johor Bahru, MYS; 3 Orthopaedics, Hospital Tuanku Ja'afar, Seremban, Seremban, MYS

**Keywords:** acetabular fracture, preoperative traction, skeletal traction, traction weight, vector traction

## Abstract

This prospective study aimed to assess the effect of gradual increment of vector traction weight on reducing displaced acetabular fractures preoperatively. Conducted at Hospital Tuanku Jaafar, Seremban, Malaysia, between June 2016 and December 2017, 21 patients with displaced acetabular fractures were included. Traction was applied using supracondylar and lateral methods, with reduction assessed by serial radiographs. Displacement was defined as greater than 2 cm in both iliopectineal and ilioischial lines. The majority of patients were male with 23 (82.1%) patients and 21 of them (75.0%) having fractures caused by road traffic accidents. Fracture reduction was achieved in 18 cases, with traction weight ranging from 5% to 33.33% of body weight. Traction applied within seven days of trauma significantly improved fracture reduction (p = 0.025). Complications, including pain and pin-tract infection, occurred in 17.9% of cases, but traction weight and duration did not significantly affect complications. The study concludes that vector traction of approximately 20% body weight effectively reduces displaced acetabular fractures preoperatively.

## Introduction

Acetabular fractures are severe injuries typically caused by high-energy trauma, such as vehicle accidents, and are often linked to multiple traumas. They are rare, with an incidence of approximately three cases per 100,000 people annually in Europe and the USA [1]. These injuries primarily affect younger men (40 years of age, 3:1 male-to-female ratio), but a secondary peak occurs in elderly patients due to low-energy fractures associated with osteoporosis [2]. Most acetabular fractures (60-75%) result from high-energy incidents such as road traffic accidents or significant falls.

Management is complex due to the anatomy of the acetabulum and patient factors. Non-displaced fractures or patients unfit for surgery are treated conservatively, while surgical intervention is necessary for displaced fractures to restore joint stability [3]. Common surgical approaches depend on fracture location, with posterior fractures accessed posteriorly, anterior fractures via an anterior approach, and complex fractures often requiring combined methods. Complications such as joint arthritis, heterotopic ossification, and nerve injuries remain prevalent despite advancements [4].

Preoperative skeletal traction, involving gradual weight increments up to 20% of body weight, is used in this center. While effective in achieving near-anatomical reduction, complications such as pin-tract infections and pressure sores require careful management [5]. Therefore, the objective of this study is to investigate the effect of pre-operative skeletal traction in reducing displaced acetabular fracture before definitive acetabular fixation surgery.

## Materials and methods

This study implemented a protocol distinct from Grubor’s approach by applying equal traction weights for the supracondylar femur and lateral traction [6]. Lateral traction was applied perpendicular to the lesser trochanter, ensuring a direct opposite force to address the medial displacement of the iliopectineal and ilioischial lines in displaced acetabular fractures.

**Figure 1:**
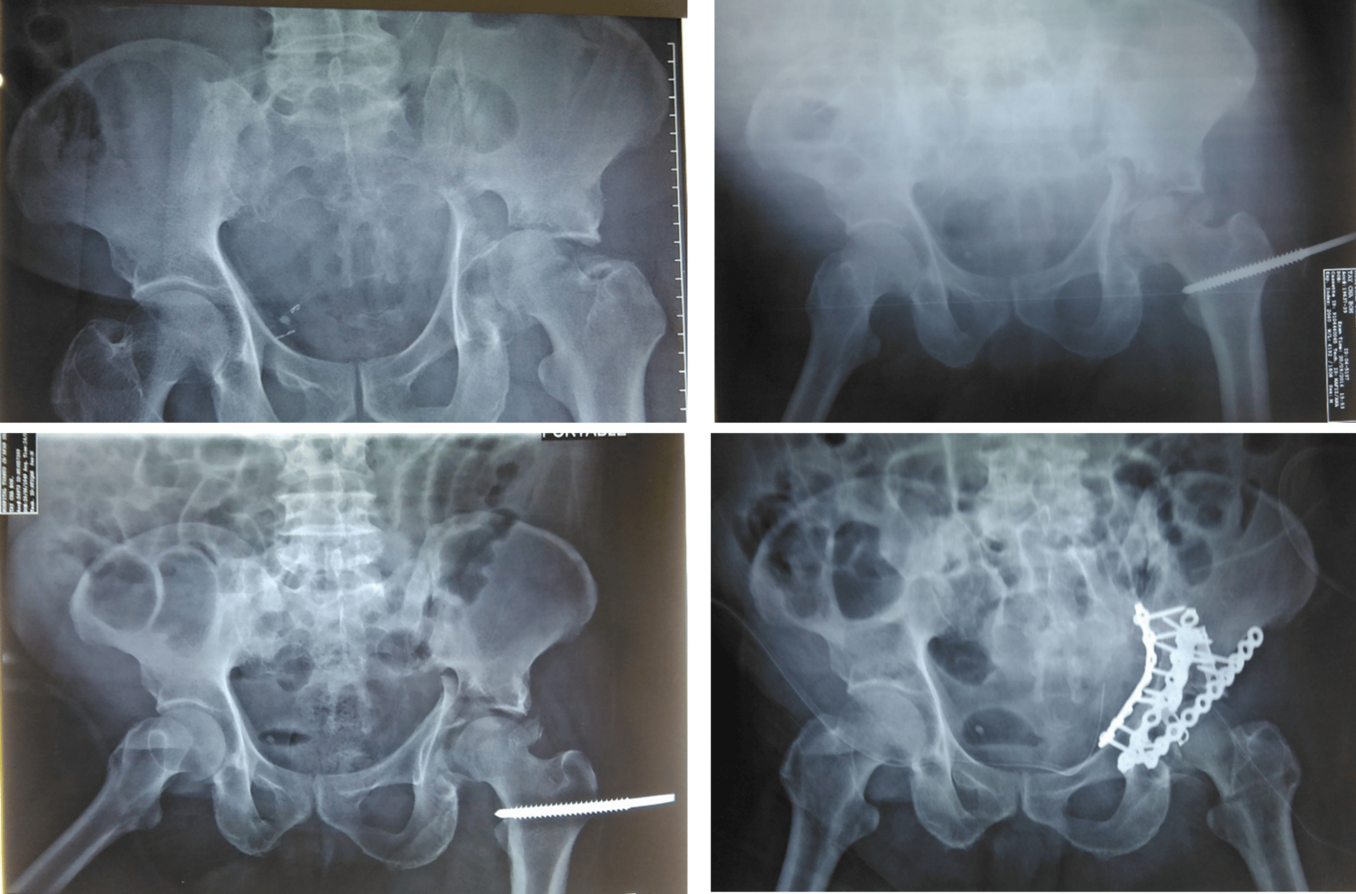
Serial radiograph of the patient before traction application with initial displacement, reduction with traction increment, and post-surgery fracture fixation

Traction weight began at 5 kg per traction, with incremental increases of 5 kg every two days until one of the following occurred: (1) acceptable fracture reduction (displacement ≤2 mm) was achieved as agreed by two trauma surgeons, (2) maximum traction weight of 30 kg was reached, (3) the surgery date arrived, (4) pain became uncontrolled (score >4) despite patient-controlled analgesia (PCA), or (5) the 13-day traction limit was reached. The reduction was assessed radiologically, and traction weight was maintained if an acceptable reduction was achieved. If no reduction occurred after 13 days, the final weight was maintained until surgery (Figure 1).

To manage pain, analgesia followed a stepped approach, starting with nonsteroidal anti-inflammatory drugs (NSAIDs) and progressing to weak opioids or PCA. Traction weight was reduced if pain exceeded tolerable levels. Strict protocols standardized radiographic techniques and reduction assessments, with trauma surgeons blinded to traction weights. Data collection included demographic, clinical, and complication-related parameters, ensuring comprehensive analysis. Withdrawal criteria allowed participants to exit without affecting their treatment

Sample size calculation

The sample size required for this study was by using the single proportion formula. The details of the calculation are expressed as below:

n=((Z_a/2_)^2^×p×q)/d^2^

Here, Z_a/2_ = 1.96, an estimated proportion of outcome conservatively treated acetabular fracture with adequate fracture reduction (good-to-excellent result vs fair-to-poor result); p = 0.83%; q = 0.17%; and d was 15%, desired absolute precision [7]. With the anticipation of a 10% non-response rate, the total number of respondents required was 27.

Statistical analysis

Data analysis was performed using Statistical Product and Service Solutions (SPSS, version 27; IBM SPSS Statistics for Windows, Armonk, NY), including demographic, descriptive, and bivariate analyses. All participant data were included in the study; for those excluded or withdrawn, only demographic data were used for descriptive purposes. Descriptive data were reported as mean ± standard deviation (SD). For normally distributed data, one-way ANOVA or independent sample T-test was applied. For non-normally distributed data, the Mann-Whitney U or Kruskal-Wallis test was used. Categorical data were analyzed with Fisher’s exact test, with a significance level set at a p value of less than 0.05.

Ethical clearance

The study was conducted in compliance with ethical principles outlined in the Declaration of Helsinki and the Malaysian Good Clinical Practice Guideline. Ethical clearance has been sought from the National Medical Research Registry, for which the study has obtained ethical clearance from the Malaysian Research Ethics Committee (MREC: 18-1625-39946 (IIR)) and KKM/NIHSEC/P18-1549(10). There are no minors involved in the study. All required information has been explained clearly in the Patient Information Sheet (PIS) and by the investigator prior to the enrolment of participants.

## Results

A total of 28 patients with displaced acetabular fractures were admitted during the recruitment period, comprising 23 males and five females, with a mean age of 41.2 years (range: 18-72 years). The mean weight was 81.2 kg (range: 50-180 kg). Of these, eight (28.6%) patients were admitted directly, while 20 (71.4%) were referrals from other centers. At presentation, one (3.6%) patient was classified as unstable, 11 (39.3%) as borderline, and the remaining majority as stable. The primary mechanism of injury was motor vehicle accidents with 21 (75%) cases, followed by falls from height with five (17.9%) cases and trivial falls with two (7.1%) cases.

Both-column fractures predominated with 13 (46.4%) cases, followed by T-type fractures with seven (25%) cases, and transverse with posterior wall fractures with four (14.3%) cases. Right-sided fractures accounted for 16 (57.1%) cases, and hip dislocations occurred in mine (32.1%) cases. Isolated acetabular fractures were observed in 11 (39.3%) cases, while the majority had associated injuries. The summary result is presented in Table 1.

**Table 1:** Demographic of respondents (N=28)

Factors	Frequency (%)
Gender	
Male	23 (82.1)
Female	5 (17.9)
Age (Mean ± standard deviation)	41.2±13.5
Race	
Malay	16 (57.1)
Chinese	8 (28.6)
Indian	2 (7.1)
Others	2 (7.1)
Admission status	
Direct admission	8 (28.6)
Referral from another center	20 (71.4)
Mechanism of injury	
Road traffic accident	21 (75.0)
Fall from height	5 (17.9)
Slip and fall	2 (7.1)
Type of acetabular fracture following Letournel classification	
Posterior wall	1 (3.6)
Transverse and Posterior wall	4 (14.3)
Anterior column and posterior hemitransverse	2 (7.1)
Transverse	1 (3.6)
T-type	7 (25.0)
Two column	13 (46.4)
Fracture side	
Right	16 (57.1)
Left	12 (42.9)
Presence of hip dislocation	
Yes	9 (32.1)
No	19 (67.9)
Other associated injuries	
Yes	17 (60.7)
No	11 (39.3)
Complications	
No complication	23 (82.1)
Pain	3 (10.7)
Pin site infection	2 (7.1)
Patient outcome	
Discharge home	26 (92.9)
Passed away	2 (7.1)

Effect of traction on fracture reduction

Out of the 28 patients, 21 (75%) were eligible for the application of both types of traction. The remaining seven patients were excluded due to various reasons: 28.6% had ipsilateral femur fractures, 14.2% refused to participate, 28.6% had severe soft tissue injuries that made traction impossible, and another 28.6% were treated with skin traction instead of skeletal traction. Therefore, among the 21 patients who underwent traction, 18 (85.7%) achieved fracture reduction. Both-column fractures demonstrated an 88.9% reduction rate with eight out of nine, while all T-type fractures achieved a reduction of 100% (6/6). However, only 33.3% (1/3) of transverse and posterior wall fractures achieved reduction. The reduction was also achieved in one case each of the anterior columns with posterior hemitransverse fractures and posterior wall fractures. Despite these differences, fracture type did not significantly affect reduction success (p = 0.081).

Traction volume requirement

Traction weights ranged from 5.0 kg to 20.0 kg, with a mean of 13.8 kg. Nine patients (42.9%) required 15.0 kg, followed by five patients (23.8%) requiring 20.0 kg. The median traction volume required was 200.0 mg/kg (range: 50.0-333.3 mg/kg), though this was not statistically significant (p = 0.740). Traction applied within seven days of trauma significantly improved reduction outcomes (p = 0.025). All patients with traction applied within seven days achieved reduction, compared to two failures among those treated within 8-14 days and one failure treated after 21 days.

Traction volume and reduction success

Seventeen patients (81%) required traction volumes exceeding 10% of their body weight, with no statistical significance between traction volume categories and reduction success (p = 0.489). Mean traction volumes per kilogram varied by fracture type: both-column fractures required 201.7 mg/kg, T-type fractures 166.3 mg/kg, and transverse/posterior wall fractures 155.2 mg/kg.

Complications

Complications were observed in five of 21 patients (17.9%), including uncontrolled pain (three cases) and pin-site infections (two cases). No pressure sores were reported. Traction weights and application duration (range: 3-13 days, median: 12 days) were not significantly associated with complications (p = 0.667).

## Discussion

Acetabular fractures, though uncommon, pose significant challenges for surgeons due to their complex anatomy and the need for specialized management strategies [8]. Displaced acetabular fractures often require definitive surgical intervention. However, early referral is difficult in developing countries due to the limited availability of centers equipped for pelvic surgery, delays in obtaining CT scans for accurate fracture evaluation, and the need for initial stabilization of patients with associated injuries.

Over 18 months, 28 patients with displaced acetabular fractures were admitted to Hospital Tuanku Jaafar Seremban, with 71.4% being referrals from another center. This reflects the hospital's role as a regional advanced trauma center catering to southern and central Peninsular Malaysia. The mean patient age was 41.2 years, older than the average of 32.5 years reported by Syed et al. but slightly younger than the average of 45 years reported by Cuthbert et al. [9,10]. Notably, 25% of patients were over 60 years old, consistent with the bimodal distribution of acetabular fractures described in recent studies, where elderly patients experience low-energy trauma [11].

Males accounted for 82.1% of cases, consistent with prior findings [9,10]. Most injuries (75%) were caused by motor vehicle accidents, with others resulting from falls from height (17.9%) or trivial falls (7.1%). Fragility fractures in elderly patients represent a growing subset of acetabular trauma, driven by increasing osteoporosis prevalence [12,13]. Right-sided fractures (57.1%) were slightly more common, and 67.9% were associated with hip dislocation, consistent with prior reports [14-16]. This pattern aligns with findings from Kandasamy et al.,which identified right-sided dominance in acetabular fractures due to trauma mechanisms such as motor vehicle accidents [17]. Similarly, Singh et al. reported a significant relationship between acetabular fractures and hip dislocations, emphasizing their coexistence as a result of high-energy impact forces [18].

Further supporting these observations, a study by Harrison et al. noted that hip dislocations commonly accompany acetabular fractures, increasing the complexity of management and the risk of complications [19]. Another analysis by Alonso et al. highlighted the poorer functional outcomes in patients with acetabular fractures involving hip dislocations compared to those without, underscoring the importance of early and effective intervention to reduce morbidity [20]. These findings collectively highlight the clinical significance of acetabular fractures, especially those associated with hip dislocations, and underscore the necessity for prompt, tailored management strategies to mitigate long-term adverse outcomes and improve patient recovery.

Fractures were classified using the Judet and Letournel system, with both-column fractures being the most common (46.4%), followed by T-type (25%) and transverse with posterior wall fractures (14.3%) [14]. This differs from other studies, where posterior wall fractures predominate [10]. Consistent with previous findings, 60.7% of patients had associated injuries, reflecting the high-energy mechanisms involved [10].

The management of fracture reduction, particularly through the application of traction, is a critical area of orthopedic practice. In this study, we analyzed the outcomes of traction for fracture reduction in a cohort of 21 patients, where 18 (85.7%) achieved successful radiological fracture reduction. The results indicate that both column fractures had an 88.9% success rate, while T-type fractures achieved complete reduction. However, transverse and posterior wall fractures exhibited significantly lower success rates at 33.3%. These findings align with previous literature suggesting that the type of fracture plays a substantial role in determining the effectiveness of traction methods.

The timing of traction application emerged as a crucial factor influencing reduction outcomes. Our results demonstrated that delayed traction application beyond seven days significantly decreased the likelihood of successful fracture reduction (p = 0.025). Early traction (within seven days) resulted in a perfect 100% reduction rate, highlighting the importance of prompt intervention in fracture management. This observation is consistent with findings from recent studies indicating that earlier intervention correlates with improved outcomes in various fracture types, including those involving the distal radius and hip joints [21,22].

Traction volumes utilized in our study ranged from 5.0 to 20.0 kg, with a mean volume of 13.81 kg. Notably, most patients (81%) required traction volumes exceeding 10% of their body weight, with effective ranges typically between 15% and 20%. Interestingly, the success of reduction was not significantly influenced by traction weight (p = 0.740) or whether it exceeded 10% of body weight (p = 0.489). This suggests that, while adequate traction is necessary, there may be an optimal range that does not strictly adhere to body weight percentages, as indicated by other studies exploring traction techniques [19,20].

Specific fracture types demonstrated varying requirements for effective traction. Both column and posterior acetabular fractures required approximately 200 mg/kg, while T-type fractures needed less at around 166 mg/kg. Transverse and posterior wall fractures required approximately 155 mg/kg, and anterior column fractures with posterior hemitransverse elements required about 188 mg/kg. These results suggest that individualized approaches based on fracture subtypes are essential for optimizing traction therapy.

Complications from traction were observed in 17.9% of cases, including pain (14.3%) and pin-site infections (9.5%). No pressure sores were reported. Pain was more common at higher traction weights, with some patients requiring patient-controlled analgesia (PCA) and reduction in traction weight. One severe pin-site infection necessitated debridement and early removal of lateral traction.

The duration of traction ranged from three to 13 days (median: 12 days). Neither traction weight nor application duration significantly influenced complication rates (p = 0.667, p = 1.000). Most patients (92.6%) were discharged without complications. However, two deaths occurred: one from sepsis related to prolonged hospitalization and another from a myocardial infarction during surgery.

Limitations

This study highlights the importance of timely traction application, with early intervention (within seven days) significantly improving reduction rates. The findings also provide practical guidance on traction volume requirements, suggesting that 15-20% of body weight is effective for most fractures.

However, limitations include the small sample size and unequal representation of fracture subtypes. Further research with larger cohorts is necessary to confirm these findings and establish more precise traction protocols for different fracture types.

## Conclusions

Acetabular fracture reduction is significantly influenced by the timing of traction application, with early traction yielding superior outcomes. Traction volumes of approximately 20% of body weight are generally effective, though specific requirements vary by fracture type. While complications from traction are infrequent, proper management of pain and pin-site care is critical. These findings underscore the need for timely referral and standardized protocols to optimize outcomes for displaced acetabular fractures, particularly in resource-limited settings.
